# Identification of Suitable Reference Genes for Gene Expression Studies in Tendons from Patients with Rotator Cuff Tear

**DOI:** 10.1371/journal.pone.0118821

**Published:** 2015-03-13

**Authors:** Mariana Ferreira Leal, Paulo Santoro Belangero, Eduardo Antônio Figueiredo, Carina Cohen, Leonor Casilla Loyola, Carlos Vicente Andreoli, Marília Cardoso Smith, Alberto de Castro Pochini, Benno Ejnisman, Moises Cohen

**Affiliations:** 1 Departamento de Ortopedia e Traumatologia, Universidade Federal de São Paulo, São Paulo, SP, Brazil; 2 Disciplina de Genética, Departamento de Morfologia e Genética, Universidade Federal de São Paulo, São Paulo, SP, Brazil; Lunenfeld-Tanenbaum Research Institute, CANADA

## Abstract

Rotator cuff tear is one of the most common causes of shoulder dysfunction. Gene expression analysis may be a useful tool for understanding tendon tears and the failure of cuff healing, and reverse-transcription quantitative polymerase chain reaction (RT-qPCR) has become an effective method for such studies. However, this technique requires the use of suitable reference genes for data normalization. Here, we evaluate the suitability of six reference genes (*18S*, *ACTB*, *B2M*, *GAPDH*, *HPRT1* and *TBP*) using samples from the rotator cuff tendons of 28 individuals with tendon tears (3 tendons regions) and 8 controls (2 tendon regions); for the tear patients, we evaluated ruptured and non-ruptured tendon samples. The stability of the candidate reference genes was determined using the NormFinder, geNorm, BestKeeper and DataAssist software packages. Overall, *HPRT1* was the best single reference gene, and *HPRT1+TBP* composed the best pair and *HPRT1+TBP+ACTB* composed the best trio of reference genes from the analysis of different groups, including the simultaneous analysis of all tissue samples. To identify the optimal combination of reference genes, we evaluated the expression of *COL1A1* and *COL3A1*, and no obvious differences were observed when using 2, 3 or 4 reference genes for most of the analyses. However, *COL3A1* expression differed between ruptured and non-ruptured (posterior superior region) tendons of patients only when normalized by *HPRT1+TBP+B2M* and *HPRT1+TBP*. On the other hand, the comparison between these two groups using the best trio of reference genes (*HPRT1+TBP+ACTB*) and 4 reference genes did not revealed a significant difference in *COL3A1* expression. Consequently, the use of suitable reference genes for a reliable gene expression evaluation by RT-qPCR should consider the type of tendon samples investigated. *HPRT1+TBP+ACTB* seems to be the best combination of reference genes for the analysis of involving different tendon samples of individuals with rotator cuff tears.

## Introduction

Rotator cuff degeneration is a very common orthopedic condition, and there are multiple factors that eventually lead to a full-thickness rotator cuff tear [1]. The incidence rate of degenerative rotator cuff tears increases with age; thus, degenerative rotator cuff tears will become an increasingly prevalent clinical problem [2]. Surgical repair of tendon tears significantly improves pain and function; however, recurrent tearing of the rotator cuff is not infrequent [2].

Several studies have been performed to elucidate the molecular alterations involved in tendon tear and the failure of cuff healing (for a review, see [1,2,3,4]). An improved understanding of the regulation of gene expression in normal and injured tendons will be important for guiding patient management and the development of new therapeutic options complementary to surgery.

As a result of its accuracy, sensitivity and capacity for high throughput analysis, reverse-transcription quantitative polymerase chain reaction (RT-qPCR) is currently considered to be the gold standard technique for evaluation of gene expression [5]; furthermore, this technique is commonly used to validate data obtained by other methods [6].

To obtain reliable data using RT-qPCR, gene expression levels must be normalized using internal controls within each sample [7]. The use of one or more reference genes can correct biases caused by variations in the complementary DNA (cDNA) input or the efficiency of reverse transcription or amplification. Ideally, reference genes should be stably expressed or at least vary only slightly in expression in all tissues or cells under the conditions of the experiment [8].

The suitability of reference genes has been evaluated in some human musculoskeletal diseases, such as shoulder instability [9], osteoarthritic articular cartilage (hip and knee) [10], human lumbar vertebral endplate with modic changes [11] and skeletal muscle with chronic degenerative changes [12]. Using a semi-quantitative approach, Lo et al. [13] described that they evaluated several widely accepted housekeeping genes (e.g., *ACTB*, *HPRT* and *GAPDH*) and observed that *GAPDH* mRNA levels are constant in dense connective tissues at different times in both normal and injured/healing tissue. Although the gene stability data were not provided, *GAPDH* [13,14,15,16,17] and *ACTB* [18,19] have been used as a reference genes in the study of mRNA regulation in human rotator cuff tear.

To our knowledge, no previous studies have clearly described the best individual or set of reference genes for gene expression analysis from samples of tendon, especially using a quantitative approach. In this study, we assessed the suitability of six reference genes frequently reported in the literature (*18S*, *ACTB*, *B2M*, *GAPDH*, *HPRT1* and *TBP*) using tendon samples from individuals with and without rotator cuff tear by analyzing gene stability with four software packages.

## Materials and Methods

### Patients

Tissue samples were obtained from 28 patients undergoing arthroscopic rotator cuff repair in the São Paulo Hospital of the Federal University of São Paulo (UNIFESP), Brazil. The following inclusion criteria were employed: age between 30 and 70 years old, the presence of full-thickness supraspinatus tears confirmed in the surgery, a minimum of 6 months of conservative treatment and no oral administration of nonsteroidal anti-inflammatory drugs within 3 days and/or no corticosteroid injection within 3 months. All patients presented a subscapular tendon without alterations by MRI and arthroscopic examination. Patients with a history of prior surgery, glenohumeral arthritis and labrum pathology were excluded from the study. In addition, we also excluded patients with tears greater than 5 cm (massive tears according to Cofield et al. [20]) and with fatty degeneration greater than grade 2 according to Fuchs et al [21].

Additionally, 8 patients operated on for proximal humeral factures were included in this study as a control group. These patients did not present any history of rotator cuff tear; furthermore, we did not find any radiological indications (X-ray and ultrasound) of this affection. All control patients were physically active. Table 1 displays the main clinical outcomes of the studied cases and controls.

**Table 1:** Distribution of the clinical outcomes of rotator cuff tear patients and controls.

**Variable**	**Cases (N = 28)**	**Controls (N = 8)**
Age at surgery, years (mean ± SD)	55 ± 8.2	55 ± 14.6
Gender (% of male)	46.4%	62.5%
Duration of condition, months (mean ± SD)	13 ± 16	-
Tear size (% of small to medium)^a^	75%	-
Mechanism (% of traumatic onset of symptoms)	25%	

^a^Tear size according to Cofield et al. [20]. N: number of samples; SD: standard deviation.

The study was approved by the ethics committee of UNIFESP. Written informed consent with approval of the ethics committee was obtained from all patients prior to specimen collection.

### Tissue samples

Specimens of about 2 mm^3^ were obtained from tendons. To collect tissue samples from the patients, we performed a standard arthroscopic rotator cuff procedure in the beach chair position under general anesthesia. The standard portals created were posterior, anterior, lateral and anterolateral. First, we performed a direct arthroscopic examination of the joint. Then, tissue samples representative of the three sectors of the rotator cuff according to Habermeyer et al. [22] were biopsied: anterior cuff (AC), posterior superior cuff (PC) and central cuff (CC), which represents the macroscopically injured supraspinatus tendon. Biopsy samples of the AC (subscapular tendon) were obtained with the scope in the posterior portal and the basket grasper in the anterior portal to represent a normal tendon of the rotator cuff. In the subacromial space, we obtained samples of the supraspinatus tendon. The CC sample (the torn supraspinatus edge) was obtained with the scope in the posterior portal and the basket grasper in the lateral portal; the most degenerated site of the tear was chosen for this biopsy. With the humerus in internal rotation, we then obtained the PC sample to represent a tendon without macroscopic alteration and presenting the insertion in the native footprint.

In the controls, a deltopectoral approach was performed to treat their fractures. Samples were obtained from the superior edge of the subscapularis tendon (anterior cuff of controls, ACC). In addition, a supraspinatus sample was obtained at insertion in the footprint (central cuff of controls, CCC).

All tissue specimens were immediately immersed in Allprotect Tissue Reagent (Qiagen, USA) and stored at −20°C until RNA extraction.

### RNA extraction

Total RNA was extracted from 10–20 mg of tissue sample using an AllPrep DNA/RNA/miRNA Mini Kit (Qiagen, USA) according to the manufacturer’s protocol. The mechanical lysis step was performed using the Tissue Lyser LT equipment (Qiagen, USA). RNA concentration and quality were immediately determined using a Nanodrop ND-1000 (Thermo Scientifc, USA) and the integrity of the RNA was verified by gel electrophoresis on a 1% agarose gel. Aliquots of the total RNA were stored at −80°C until further use.

### RT-qPCR

RT-qPCR gene expression quantifications were performed according to MIQE guidelines [23]. Only RNA samples with the optical density (OD)_260/280_ > 1.8 were used, following the MIQE protocol.

First, cDNA was synthesized from 150 ng of RNA using a High-Capacity cDNA Reverse Transcription Kit (Life Technologies, USA) according to the manufacturer’s protocol.

To detect the range of expression of the six candidate reference genes, reactions were performed with 52.5 ng of cDNA input using TaqMan Low-Density Array (TLDA) cards (Life Technologies, USA) and ViiA 7 Real-Time PCR System (Life Technologies, USA). Only inventoried TaqMan Gene Expression Assays (Life Technologies, USA) were choose for gene expression analysis. The final volume in each TLDA well is approximately 1 μl. All reactions were performed in triplicate.

To identify the best combination of reference genes, we also quantified the mRNA expression of target genes, *COL1A1* and *COL3A1*, using the candidate reference genes for normalization. The upregulation of *COL1A1* and *COL3A1* have been reported in several joint injuries, including rotator cuff tear [13,18], injured Achilles tendon [3,24], anterior cruciate ligament [25,26,27] and glenohumeral capsule of shoulder instability [28].

For each sample, the candidate reference and target genes were assayed on the same card to exclude technical variations. The 6 reference genes and target gene are summarized in Table 2.

**Table 2:** Summary of six reference genes and target genes.

**Gene symbol**	**Name**	**Gene function**	**Assay** *
*18S*	Eukaryotic 18S rRNA	Ribosome subunit	Hs99999901_s1
*ACTB*	Beta-actin	Cytoskeletal structural protein	Hs01060665_g1
*B2M*	Beta-2-microglobulin	Beta-chain of major histocompatibility complex class I molecules	Hs00984230_m1
*GAPDH*	Glyceraldehyde-3-phosphate dehydrogenase	Oxidoreductase in glycolysis and gluconeogenesis	Hs02758991_g1
*HPRT1*	Hypoxanthine phosphoribosyl-transferase	Purine synthesis in salvage pathway	Hs02800695_m1
*TBP*	TATA box binding protein	RNA polymerase II, transcription factor	Hs00427620_m1
*COL1A1*	Collagen, type 3, alpha 1	Extracellular matrix structural protein	Hs00164004_m1
*COL3A1*	Collagen, type 3, alpha 1	Extracellular matrix structural protein	Hs00943809_m1

*TaqMan probes were purchased as assays-on-demand products for gene expression (Life Technologies, USA).

The relative threshold method (Crt method) was applied, which is a robust method that sets a threshold for each curve individually based on the shape of the amplification curve, regardless of the height or variability of the curve during its early baseline fluorescence. The expression of collagen genes across the samples was calculated using the equation ΔCrt, in which [ΔCrt = target gene (collagen) Crt—the mean of reference genes Crt].

### Analysis of reference gene expression stability

We categorized the tissue samples into the following 12 groups: 1) CC samples (N = 28); 2) PC samples (N = 28); 3) AC samples (N = 28); 4) CC and PC samples (N = 56); 5) CC and AC samples (N = 56); 6) all tissue samples from cases (N = 84); 7) CCC samples (N = 8); 8) ACC samples (N = 8); 9) all tissue samples from controls (N = 16); 10) CC and CCC samples (N = 36); 11) AC and ACC samples (N = 36) and 12) all tissue samples (N = 100). Typically, gene expression studies compare transcript levels between case (i.e., the injured tissue) and control samples. We considered three types of possible controls: PC, AC [15] and CCC [13,17,18] samples; therefore we created the groups #4, #5 and #10, respectively. However, some researchers have been investigated a possible association between gene expression and clinical variables [14,19] and also have been reported the presence of molecular alterations of subscapular tendon samples of patients with supraspinatus tears [14,15]; therefore we chose to present the analysis of suitable reference gene in each type of tendon. In addition, the group composed by all tissue samples from controls (group #9), as well as was the group composed by macroscopically non-injured subscapular tendons (group #11), were created since the understanding of gene expression regulation in non-injured tendons is still necessary.

For comparisons of candidate reference gene stability we used NormFinder (http://www.mdl.dk/publicationsnormfinder.htm), geNorm (http://medgen.ugent.be/∼jvdesomp/genorm/http://medgen.ugent.be/∼jvdesomp/genorm/), BestKeeper1 (http://www.gene-quantifcation.de/bestkeeper.html) and DataAssist (http://www.lifetechnologies.com/us/en/home/technical-resources/software-downloads/dataassist-software.html) software programs according to the recommendations of the software guides.

NormFinder accounts for both intra- and inter-group variations when evaluating the stability of each single reference gene [29]. The stability values and standard errors are calculated according to the transcription variation of the reference genes. Stably expressed genes, which have low variation in expression levels, present low stability values. NormFinder analysis also calculated the stability value for two reference genes.

geNorm calculates the expression stability value (M) for each gene based on the average pairwise expression ratio between a particular gene and all other reference genes. geNorm sequentially eliminates the gene that shows the highest variation relative to all the other genes based on paired expression values in all the studied samples. The most stably expressed gene yields the lowest M value, and then the two most stable reference genes are determined by stepwise exclusion of the least stable gene [7]. Because of the elimination process, geNorm cannot identify a single suitable reference gene, and ends up by suggesting a pair of genes that shows high correlation and should be suitable for normalization of qPCR studies.

Bestkeeper was used to rank the 6 reference genes based on the standard deviation (SD) and coefficient of variance (CV) expressed as a percentage of the cycle threshold (Ct) level [30]. The more stable reference gene presents the lowest CV and SD. Bestkeeper also uses a statistical algorithm wherein the Pearson correlation coefficient for each candidate reference gene pair is calculated along with the probability of correlation significance of the pair.

Lastly, DataAssist software provided a metric to measure reference gene stability based on the geNorm algorithm. In contrast to the other programs, DataAssist uses RQ to calculate the stability value of individual candidate reference genes. The lower score represents the more stable the control.

GenEx software (http://genex.gene-quantifcation.info/) was used to determine the optimal number of reference genes by calculating the accumulated standard deviation (Acc.SD). If larger number of reference genes is used, random variation among the genes’ expression partially cancel reducing the SD. A minimum in the Acc.SD plot indicate the number of reference genes that give the lowest SD.

### Statistical analysis

To compare collagen expression between the groups, we first verified the distribution of the data using the Shapiro-Wilk normality test for the determination of the appropriate tests for the subsequent statistical comparisons. ΔCrt was normally distributed, with the exception that collagen expression was normalized by only *GAPDH* in some groups of samples. Therefore, the paired T-test and independent T-test were performed to compare gene expression between the studied groups. A p-value of < 0.05 was considered statistically significant. All values are shown as the mean ± standard deviation (SD).

## Results

### Reference gene expression levels

**Fig 1:**
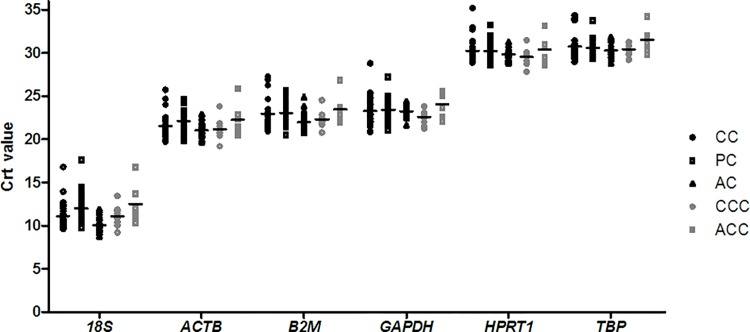
RT-qPCR detection of the expression levels of six reference genes. A lower cycle threshold value (Crt) indicates higher gene expression. CC: central cuff (injured supraspinatus tendon) samples of cases; PC: posterior superior cuff samples of cases; AC: anterior cuff samples of cases; CCC: central cuff samples of controls; ACC: anterior cuff samples of controls.

The distribution of Crt values for each of the 6 candidate reference genes is shown in Fig. 1. These genes displayed a wide range of expression levels. *18S* presented the highest expression level among the candidate reference genes (mean Crt value ± SD: 11.14 ± 1.61). In contrast, *TPB* (30.57 ± 1.06) and *HPRT1* (30.06 ± 1.11) presented the lowest expression level in the rotator cuff tendon samples.

### Reference gene expression stability

S1 Table displays the stability value ranking of the single candidate reference genes as determined by the different software packages. In our analysis, all the reference genes for all the analysis groups presented M values less than the geNorm threshold of 1.5, which is recognized as stable (S1 Table). However, *18S* and *B2M* presented high SD of Crt in the analysis involving all samples according to BestKeeper software (SD = 1.17, SD = 1.03, respectively), in which any studied gene with the SD higher than 1 can be considered inconsistent.

Although none of the four software packages suggested the same best reference genes in the studied sample groups, the software packages did generate similar rankings of reference gene stability for each analysis group (S1 Table).

Table 3 shows the best suitable reference gene by the different software packages. In the present study, *HPRT1*, followed by *TBP*, was the most suitable reference gene for the studied of tendons of the rotator cuff.

**Table 3:** Best reference gene for each group of sample.

**Groups**	**Best reference gene by software**			
	**NormFinder**	**geNorm**	**BestKeeper**	**DataAssist**
**Samples of cases**				
CC	***HPRT1***	***HPRT1*** or ***ACTB***	*TBP*	***ACTB***
PC	***HPRT1***	***HPRT1*** or ***TBP***	***TBP***	***HPRT1***
AC	***ACTB***	***HPRT1*** or ***ACTB***	***HPRT1***	***ACTB***
CC and PC	***HPRT1***	***HPRT1*** or ***TBP***	***TBP***	***HPRT1***
CC and AC	***ACTB***	*HPRT1* or ***ACTB***	*TBP*	***ACTB***
All samples of cases	***HPRT1***	***HPRT1*** or ***TBP***	***TBP***	***HPRT1***
**Samples of controls**				
CCC	***TBP***	***HPRT1*** or *B2M*	***TBP***	***HPRT1***
ACC	***TBP***	***HPRT1*** or ***TBP***	***TBP***	***HPRT1***
All samples of controls	***TBP***	*B2M* or *ACTB*	***TBP***	***TBP***
**Samples of cases and controls**				
CC and CCC	***HPRT1***	***HPRT1*** or *ACTB*	*TBP*	***HPRT1***
AC and ACC	*TBP*	*B2M* or ***ACTB***	*HPRT1*	***ACTB***
All samples	***HPRT1***	***HPRT1*** or ***TBP***	***TBP***	***HPRT1***

CC: central cuff (injured supraspinatus tendon) samples of cases; PC: posterior superior cuff samples of cases; AC: anterior cuff samples of cases; CCC: central cuff samples of controls; ACC: anterior cuff samples of controls; Bold letters: best pairs of reference genes by more than one software.

As previously described, gene expression studies typically compare transcript levels between injured and non-injured tissue samples. When the CC and PC samples were evaluated together, *HPRT1*, followed by *TBP*, was the most suitable reference gene. When considering the CC and AC samples, NormFinder, geNorm and DataAssist each identified *ACTB*, followed by *HPRT1*, as the most stable gene (Table 3; S1 Table). In contrast, these three software packages identified *HPRT1*, followed by *ACTB*, as the most stable gene in the analysis involving the CC and CCC samples (Table 3; S1 Table).

When we individually evaluated each tendon region of patients with rotator cuff tears, we observed that *HPRT1* was the most stable gene for the CC and PC samples. *ACTB* was identified as the most stable gene by NormFinder and DataAssist software for the AC samples, whereas geNorm and BestKeeper identified *HPRT1* as the most stable gene in this group of samples. When all samples of the cases were considered, *HPRT1* was identified as the most stable gene by NormFinder, geNorm and DataAssist.

In the analyses involving only tissue samples from the control individuals, we observed that *TPB* and *HPRT1* were the most stable genes. However, *ACTB* was identified as the most stable genes in the analysis involving macroscopically non-injured subscapular tendons.

### Analysis of the best combinations of reference genes

Table 4 displays the best combinations of reference genes, as suggested by the software packages. Overall, *HPRT1* and *TBP* were the most suitable reference genes, and this pair of genes was the one most frequently identified when evaluating all samples of the cases or all tissue samples, as well as when evaluating samples of (1) CC and PC, (2) PC and (3) ACC. In contrast, *HPRT1* + *ACTB* was the most frequently identified pair from the analysis of samples of (1) CC, (2) AC, (3) CC and AC and (4) CC and CCC. In addition, *HPRT1* + *B2M* was the most frequently identified pair of reference genes from the analysis of CCC samples. Moreover, *ACTB* + *B2M* was the most frequently identified pair from the analysis of all controls and of the group composed by the AC and ACC samples.

**Table 4:** Best combination of reference genes for each group of sample.

**Groups**	**Best pair of reference genes by software**				**Best trio of reference genes^a^**
	**NormFinder**	**geNorm**	**BestKeeper**	**DataAssist**	
**Samples of cases**					
CC	***HPRT1+ACTB***	***HPRT1+ACTB***	***HPRT1+ACTB***	*HPRT1*+*TBP*	*ACTB+HPRT1+TBP*
PC	***HPRT1*+*TBP***	***HPRT1*+*TBP***	*HPRT1+B2M*	***HPRT1*+*TBP***	*B2M+HPRT1+TBP*
AC	*ACTB*+*TBP*	***HPRT1+ACTB***	*ACTB*+*B2M*	***HPRT1+ACTB***	*ACTB+HPRT1+TBP*
CC and PC	*ACTB*+TBP*	***HPRT1*+*TBP***	***HPRT1*+*TBP***	***HPRT1*+*TBP***	*ACTB+HPRT1+TBP*
CC and AC	*ACTB*+TBP*	***HPRT1+ACTB***	***HPRT1+ACTB***	*HPRT1*+*TBP*	*ACTB+HPRT1+TBP*
All samples of cases	HPRT1+ACTB*	***HPRT1*+*TBP***	*ACTB*+*B2M*	***HPRT1*+*TBP***	*ACTB+HPRT1+TBP*
**Samples of controls**					
CCC	*HPRT1*+*TBP*	***HPRT1+B2M***	*ACTB*+*B2M*	***HPRT1+B2M***	*B2M+HPRT1+TBP*
ACC	***HPRT1*+*TBP***	***HPRT1*+*TBP***	***HPRT1*+*TBP***	***HPRT1*+*TBP***	*ACTB+HPRT1+TBP* or *B2M+HPRT1+TBP*
All samples of controls	*HPRT1*+TBP*	***ACTB*+*B2M***	***ACTB*+*B2M***	***ACTB*+*B2M***	*ACTB+HPRT1+TBP* or *B2M+HPRT1+TBP*
**Samples of cases and controls**					
CC and CCC	*HPRT1*+TBP*	***HPRT1+ACTB***	***HPRT1+ACTB***	***HPRT1+ACTB***	*ACTB+HPRT1+TBP*
AC and ACC	*ACTB*+TBP*	***ACTB*+*B2M***	***ACTB*+*B2M***	***ACTB*+*B2M***	*B2M+HPRT1+TBP*
All samples	*ACTB*+TBP*	***HPRT1*+*TBP***	*ACTB*+*B2M*	***HPRT1*+*TBP***	*ACTB+HPRT1+TBP*

^a^Best trio combination is based in a visual inspection of all the ranks generated by the four software.

*Best combination of two genes determined considering the intragroup and intergroup variation. CC: central cuff (injured supraspinatus tendon) samples of cases; PC: posterior superior cuff samples of cases; AC: anterior cuff samples of cases; CCC: central cuff samples of controls; ACC: anterior cuff samples of controls; Bold letters: best pairs of reference genes by more than one software.

The 4 software packages only indicated up to 2 genes as the best combination of reference genes. By visual inspection of all the ranks generated by the four software, we observed that *ACTB + HPRT1 + TBP* was more frequently the best trio of reference genes. In addition, *ACTB + HPRT1 + TBP* was the best trio in all the analyses involving injured and non-injured tendon samples.

**Fig 2:**
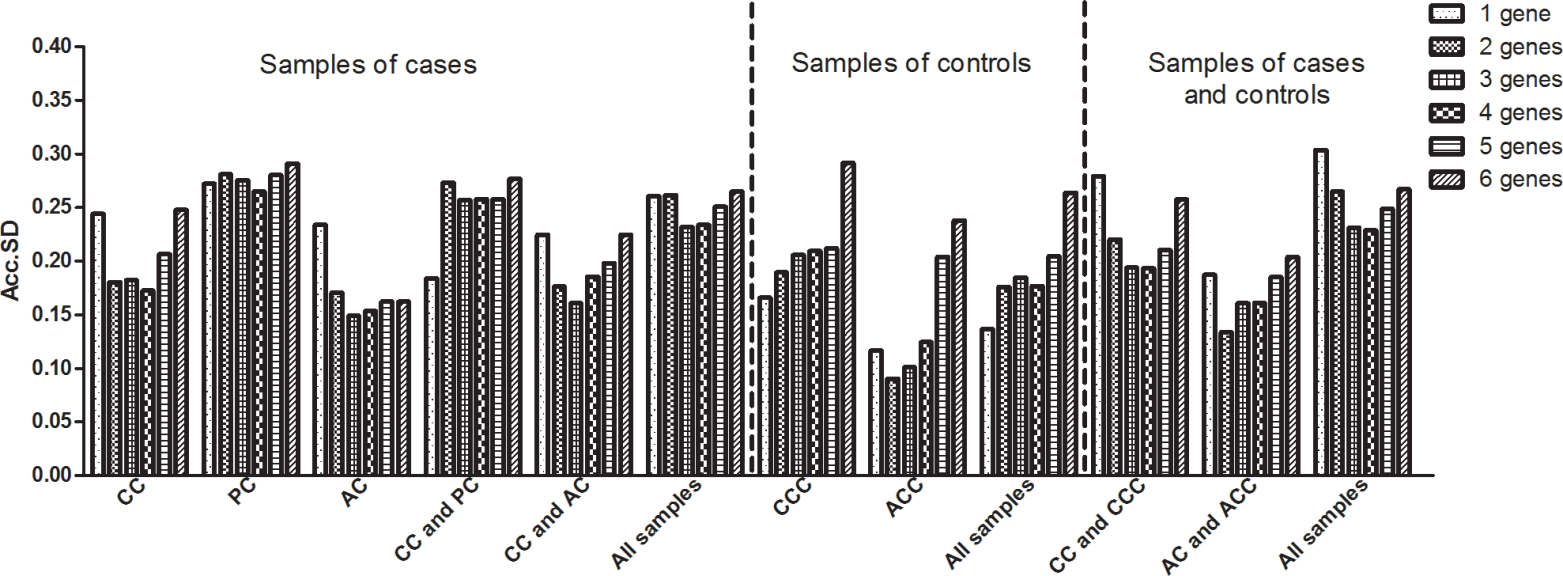
Accumulated standard deviation for the 6 reference genes in tendon samples. Lower values of accumulated standard deviation (Acc.SD) indicate the optimal number of reference gene as estimated by the GenEx software package. CC: central cuff (injured supraspinatus tendon) samples of cases; PC: posterior superior cuff samples of cases; AC: anterior cuff samples of cases; CCC: central cuff samples of controls; ACC: anterior cuff samples of controls.

We used the GenEx software package to determine whether reliable normalization would require more than 2 reference genes. In this analysis, the optimal number of reference genes is indicated by the lowest SD, and with the exception of the analysis of the CCC or ACC samples (external control tendon samples), the Acc.SD of 2 reference genes did not differ more than 0.1 from the observed metric when using more than 2 genes (Fig. 2). However, in the analysis of the CCC samples, the use of 6 reference genes presented a higher Acc.SD than that observed with the use of 2–4 reference genes. Moreover, in the ACC samples, the use of 2 or 3 genes presented the lowest Acc.SD (0.091 and 0.101, respectively).

### Effects of reference gene choice

To evaluate the effect of appropriate reference genes selection, an expression analysis was performed by comparing data from the macroscopically injured region and three possible control regions (PC, AC and CCC samples) and by comparing data from macroscopically non-injured subscapular tendon samples from both the cases and controls and comparing the tendons of the controls. This analysis was performed using *COL1A1* and *COL3A1* as target genes in all the analyses. As reference genes, we used the most frequently identified pairs described above. We also performed the collagen genes expression analysis using 3 reference genes (*HPRT1* + *TBP* + *ACTB* and *HPRT1* + *TBP* + *B2M*), 4 reference genes *(HPRT1* + *B2M* + *ACTB* + *TBP*) and only *ACTB* [18,19] or *GAPDH* [13,14,15,16,17], as previously described in the literature.

**Fig 3:**
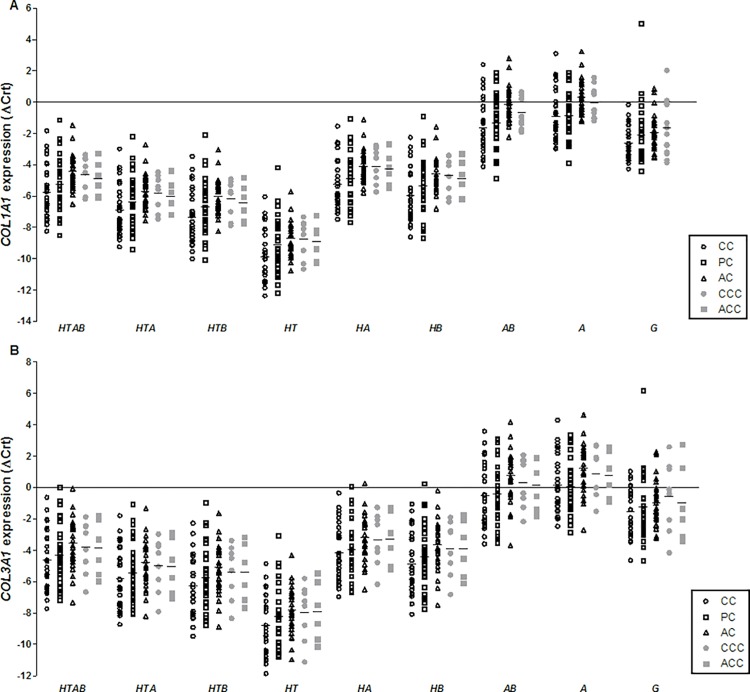
*COL1A1* (A) and *COL3A1* (B) expression normalized by different combinations of candidate reference genes in tendons specimens. HTAB: collagen expression normalized by *HPRT1 + TBP + ACTB + B2M*; HTA: collagen expression normalized by *HPRT1 + TBP + ACTB*; HTB: collagen expression normalized by *HPRT1 + TBP + B2M*; HT: collagen expression normalized by *HPRT1 + TBP*; HA: collagen expression normalized by *HPRT1 +* ACTB; HB: collagen expression normalized by *HPRT1 + B2M*; AB: collagen expression normalized by *ACTB + B2M*; A: collagen expression normalized by *ACTB*; G: collagen expression normalized by *GAPDH*; CC: central cuff (injured supraspinatus tendon) samples of cases; PC: posterior superior cuff samples of cases; AC: anterior cuff samples of cases; CCC: central cuff samples of controls; ACC: anterior cuff samples of controls.

Although the normalized expression quantities differed between the various combinations of reference genes, the distributions of collagen genes expression in the studied samples were similar (Fig. 3). By visual inspection, the distribution was slight different only when *GAPDH* was used for gene expression normalization.

*COL1A1* expression was significantly increased in the CC samples compared to the AC samples of cases when using all the reference genes combinations (p < 0.05, Table 5). However, a significant difference between the CC and AC samples was not detected when *COL1A1* expression was normalized by only *GAPDH* (p = 0.181, Table 5).

**Table 5:** *COL1A1* expression normalized by different combinations of reference genes in the rotator cuff tendons samples.

	*COL1A1* expression (ΔCrt; mean ± SD)														
Reference genes	CC	PC	p-value^a^	CC	AC	p-value^a^	CC	CCC	p-value^b^	AC	ACC	p-value^b^	CCC	ACC	p-value^a^
*HPRT1*+*TBP+ACTB*+*B2M*	−4.79±1.94	−4.33±2.01	0.182	−4.79±1.94	03.35±1.38	0.006*	−4.79±1.94	−3.85±1.66	0.221	03.35±1.38	−3.34±1.82	0.465	−3.85±1.66	−3.84±1.82	0.995
*HPRT1*+*TBP+ACTB*	−5.92±1.88	−5.47±1.95	0.172	−5.92±1.88	−4.62±1.34	0.008*	−5.92±1.88	−5.03±1.67	0.233	−4.62±1.34	−4.98±1.82	0.575	−5.03±1.67	−4.98±1.82	0.962
*HPRT1*+*TBP+B2M*	−6.40±1.98	−5.78±2.09	0.087	−6.40±1.98	−4.95±1.40	0.007*	−6.40±1.98	−5.42±1.72	0.213	−4.95±1.40	−5.39±1.91	0.518	−5.42±1.72	−5.39±1.91	0.975
*HPRT1*+*TBP*	−8.90±1.91	−8.22±2.06	0.051	−8.90±1.91	−7.64±1.35	0.012*	−8.90±1.91	−7.98±1.77	0.227	−7.64±1.35	−7.87±1.97	0.725	−7.98±1.77	−7.87±1.97	0.919
*HPRT1+ACTB*	−4.30±1.82	−4.02±1.84	0.363	−4.30±1.82	−3.00±1.31	0.005*	−4.30±1.82	−3.33±1.67	0.187	−3.00±1.31	−3.24±1.75	0.701	−3.33±1.67	−3.24±1.75	0.924
*HPRT1+B2M*	−5.01±1.97	−4.47±2.07	0.135	−5.01±1.97	−3.49±1.41	0.004*	−5.01±1.97	−3.92±1.73	0.263	−3.49±1.41	−3.85±1.88	0.599	−3.92±1.73	−3.85±1.88	0.945
*ACTB* + *B2M*	−0.68±2.00	−0.44±1.99	0.503	−0.68±2.00	0.93±1.42	0.003*	−0.68±2.00	0.28±1.59	0.222	0.93±1.42	0.19±1.67	0.272	0.28±1.59	0.19±1.67	0.918
*ACTB*	0.04±1.86	0.01±1.80	0.922	0.04±1.86	1.42±1.33	0.004*	0.04±1.86	0.87±1.56	0.258	1.42±1.33	0.80±1.55	0.319	0.87±1.56	0.80±1.55	0.934
*GAPDH*	−1.68±1.42	−1.22±2.19	0.235	−1.68±1.42	−0.89±1.41	0.181	−1.68±1.42	−0.57±2.28	0.101	−0.89±1.41	−0.96±2.47	0.914	−0.57±2.28	−0.96±2.47	0.764

^a^p- value by paired T-test

^b^p-value by independent T-test

*p < 0.05. SD: standard deviation; CC: central cuff (injured supraspinatus tendon) samples of cases; PC: posterior superior cuff samples of cases; AC: anterior cuff samples of cases; CCC: central cuff samples of controls; ACC: anterior cuff samples of controls.

*COL3A1* expression was significantly increased in the CC samples compared to the AC samples of cases when using all the reference genes combinations described above (p < 0.05, Table 6). However, a significant difference between the CC and PC samples was only detected when *COL3A1* expression was normalized by *HPRT1 + TBP* (p = 0.02; Table 6), *HPRT1 + TBP + B2M* (p = 0.042, Table 6) and only *HPRT1* (p = 0.034; data not shown) or *TBP* (p = 0.016; data not shown), which were the most stable reference genes.

**Table 6:** *COL3A1* expression normalized by different combinations of reference genes in the rotator cuff tendons samples.

	*COL3A1* expression (ΔCrt; mean ± SD)														
Reference genes	CC	PC	p-value^a^	CC	AC	p-value^a^	CC	CCC	p-value^b^	AC	ACC	p-value^b^	CCC	ACC	p-value^a^
*HPRT1*+*TBP+ACTB*+*B2M*	−5.75±1.67	−5.22±1.75	0.101	−5.75±1.67	−4.44±1.10	<0.001*	−5.75±1.67	−4.62±1.14	0.082	−4.44±1.10	−4.89±1.12	0.373	−4.62±1.14	−4.89±1.12	0.201
*HPRT1*+*TBP+ACTB*	−6.88±1.59	−6.37±1.68	0.086	−6.88±1.59	−5.71±1.07	<0.001*	−6.88±1.59	−5.79±1.15	0.083	−5.71±1.07	−6.04±1.10	0.500	−5.79±1.15	−6.04±1.10	0.181
*HPRT1*+*TBP+B2M*	−7.36±1.71	−6.67±1.85	0.042*	−7.36±1.71	−6.03±1.12	<0.001*	−7.36±1.71	−6.19±1.20	0.080	−6.03±1.12	−6.44±1.19	0.434	−6.19±1.20	−6.44±1.19	0.222
*HPRT1*+*TBP*	−9.86±1.61	−9.11±1.80	0.020*	−9.86±1.61	−8.72±1.08	0.001*	−9.86±1.61	−8.75±1.26	0.081	−8.72±1.08	−8.93±1.20	0.689	−8.75±1.26	−8.93±1.20	0.211
*HPRT1+ACTB*	−5.26±1.54	−4.91±1.58	0.219	−5.26±1.54	−4.09±1.04	<0.001*	−5.26±1.54	−4.10±1.23	0.057	−4.09±1.04	−4.30±1.06	0.660	−4.10±1.23	−4.30±1.06	0.202
*HPRT1+B2M*	−5.97±1.71	−5.36±1.83	0.078	−5.97±1.71	−4.58±1.13	<0.001*	−5.97±1.71	−4.69±1.19	0.055	−4.58±1.13	−4.90±1.19	0.534	−4.69±1.19	−4.90±1.19	0.259
*ACTB* + *B2M*	−1.64±1.76	−1.32±1.75	0.364	−1.64±1.76	−0.16±1.15	<0.001*	−1.64±1.76	−0.49±1.08	0.090	−0.16±1.15	−0.86±1.06	0.180	−0.49±1.08	−0.86±1.06	0.219
*ACTB*	−0.92±1.59	−0.88±1.53	0.883	−0.92±1.59	0.33±1.07	<0.001*	−0.92±1.59	0.10±1.05	0.099	0.33±1.07	−0.26±0.95	0.224	0.10±1.05	−0.26±0.95	0.196
*GAPDH*	−2.64±1.03	−2.11±1.92	0.115	−2.64±1.03	−1.97±1.13	0.001*	−2.64±1.03	−1.34±1.95	0.108	−1.97±1.13	−2.02±1.58	0.935	−1.34±1.95	−2.02±1.58	0.208

^a^p- value by paired T-test

^b^p-value by independent T-test

*p < 0.05. SD: standard deviation; CC: central cuff (injured supraspinatus tendon) samples of cases; PC: posterior superior cuff samples of cases; AC: anterior cuff samples of cases; CCC: central cuff samples of controls; ACC: anterior cuff samples of controls.

## Discussion

RT-qPCR is one of the most commonly utilized approaches in functional genomics research, and its use in gene expression analysis may become routine. However, many authors do not critically evaluate their RT-qPCR experiments, and as a result, the experiments are improperly designed and difficult to repeat due to insufficient data quality [31]. To minimize the influence of differences between samples in the extraction of mRNA, reverse transcription and PCR [32], is necessary to normalize target gene expression by a known factor. Consequently, the use of suitable reference genes with stable expression in the studied tissue (normal and/or injured) is essential for effective data normalization and the acquisition of accurate and meaningful biological data.

Reference genes have been described for RT-qPCR studies in several diseases and tissues [10,11,12,33,34,35,36,37], and our group recently identified the most stable reference genes in the glenohumeral capsule of patients with and without shoulder instability [9]. To the best of our knowledge, no prior study has aimed to identify suitable reference genes for gene expression analyses by quantitative approaches in human tendons.

In the present study, we used 4 software packages (NormFinder, geNorm, BestKeeper and DataAssist) to evaluate the stability of reference gene expression. As each software package implements distinct algorithms, different results can be expected. Therefore, it is important to use more than one software package to identify the most suitable reference genes among a set of candidates. Although the 4 software packages differed in their rankings of reference gene stability as well as in the identity of the most suitable pair, at least two programs produced results that agreed for almost all the analyses. Our results demonstrate that the use of 4 statistical tools aids in the identification of the best reference genes.

All the reference genes in this study presented an M value less than the geNorm threshold of 1.5 recognized as stable under the different experimental conditions tested. However, *18S* and *B2M* presented high SD of Crt in the analysis involving all samples according to BestKeeper software. Therefore, these 2 reference genes should not be used in analysis involving different types and conditions (injured and non-injured) of tendon of rotator cuff.

In the different groups of analyses, *HPRT1* appeared to be the most suitable gene overall; however, it is increasingly clear that in most situations, a single reference gene is not sufficiently stable [38]. When a larger number of reference genes is used, the SD of the normalization factor (mean of reference gene expression) is reduced and the random variation among the expression of the tested genes is partially cancelled.

Using the GenEx software, we observed that the Acc.SD value of 2 reference genes differed by no more than 0.1 from that observed when 3, 4, 5 or 6 reference genes were used in most of the analysis groups. As the inclusion of additional reference genes increases the time and money required for the analysis, it is important to consider the degree of improvement and overall noise contributed by reference genes when deciding how many reference genes are required. Considering that the reproducibility of real-time PCR equipment is rarely less than 0.1 cycle (estimated as the SD of technical replicates), we believe that the use of several reference genes does not significantly improve the data quality. However, we observed that the use of 1, 2 or 3 reference genes may lead to differences in the statistical analysis result of some group comparisons.

Although different combinations of reference genes were determined as being the most suitable for the various analysis groups, the combination of *HPRT1* + *TBP* was the most frequently identified pair and *HPRT1* + *TBP* + *ACTB* was frequently identified trio. Furthermore, our results demonstrated that these combinations of reference genes can be used in most of the comparisons between samples of injured and non-injured tendons from patients with and without rotator cuff tears.

To identify the best combination of reference genes, we evaluated *COL1A1* and *COL3A1* expression in samples of injured and non-injured tissue from the cases and controls. The statistical comparison revealed that *COL1A1* and *COL3A1* expression differed between the ruptured tendon and AC samples from the cases, independently of which reference gene combination was used for normalization. However, a significant difference between the CC and AC samples was not detected when *COL1A1* expression was normalized by only *GAPDH*.

Interestingly, *COL3A1* expression was significantly different between the ruptured and PC regions of the cases only when it was normalized by *HPRT1 + TBP*, *HPRT1 + TBP + B2M*, *HPRT1* alone or *TBP* alone, which were the most stable reference genes. Although previous studies demonstrated the involvement of *COL3A1* in rotator cuff tear [13,18], we believe that further investigations are still necessary to understand the role of this gene in rotator cuff diseases. The increased *COL3A1* expression in the ruptured tissue may be a result of the use of not suitable reference genes for data normalization. It is important to highlight that the best trio of reference genes for the comparison between CC and PC samples was *HPRT1 + TBP + ACTB*. The statistical comparison between these two groups of samples using the best trio, as well as four reference genes (*HPRT1* + *TBP + ACTB* + *B2M*), showed that *COL3A1* did not differ between CC and PC samples.

Moreover, within the case and control groups, no obvious differences in *COL1A1* and *COL3A1* expression were observed when normalized with different combinations of reference genes. The number of samples available for the independent T-test was reduced, especially the control group. However, to the best of our knowledge, no prior study evaluated RNA expression in non-cadaveric human tendon samples of a control group without tendon injuries in a study of molecular aspects involved in rotator cuff tears.

We also evaluated the effect of the use of different combinations of reference genes in the expression of other extracellular matrix genes. The different normalizations resulted in the same finding concerning the statistical comparison between groups of tendons (data not show).

Our study presented some limitations. First, we only included a limited number of candidate reference genes, and it is likely that some other genes may also be used as internal references for gene expression studies in tendon samples from patients with or without history of rotator cuff tear. Second, our results only apply directly to rotator cuff tendons. It is unclear how well our results would extend to other joint tendons. Therefore, when new cohorts of tissue samples are used, we suggest performing specific gene expression studies to identify the most stable reference genes to be used for normalization. However, it is important to highlight that our results may be relevant to the study of rotator cuff tear, as well as to the study of normal tendons.

### Conclusions

In the present study, we evaluated the suitability of reference genes using samples of tendons from individuals with and without a history of rotator cuff tear. We observed that the use of suitable reference genes for a reliable gene expression evaluation by RT-qPCR should consider the type of tendon samples investigated. Examining the different analysis groups, *HPRT1*, followed by *TBP*, appears to be the most suitable reference gene. *HPRT1* + *TBP* + *ACTB* seems to be the best combination of reference genes for the analysis of involving different tendon samples of individuals with rotator cuff tears. The results of this work may benefit future studies of shoulder human tendons that require more accurate gene expression quantification.

## Supporting Information

S1 Table Ranking of the candidate single reference genes by each software package used. (DOCX)
