# Supplementary material for: Identification of Suitable Reference Genes for Gene Expression Studies in Tendons from Patients with Rotator Cuff Tear
Source: PLoS One. 2015 Mar 13;10(3):e0118821. doi: 10.1371/journal.pone.0118821 (PMC4358921; doi:10.1371/journal.pone.0118821)
Supplement: S1 Table — (DOCX) [file pone.0118821.s001.docx]

Table S1. Ranking of the candidate single reference genes by each software package used.

| **NormFinder** | |  | **geNorm** | |  | **BestKeeper** | |  | **DataAssist** | |
| --- | --- | --- | --- | --- | --- | --- | --- | --- | --- | --- |
| **Stability value*** | **Ranking** |  | **M value*** | **Ranking** |  | **CV*** | **Ranking** |  | **Score*** | **Ranking** |
| CC samples | | | | | | | | | | |
| 0.244 | ***HPRT1*** |  | 0.462 | ***HPRT1*** |  | 2.79 | *TBP* |  | 0.6651 | ***ACTB*** |
| 0.266 | *ACTB* |  | 0.462 | ***ACTB*** |  | 3.23 | *HPRT1* |  | 0.669 | *HPRT1* |
| 0.411 | *18S* |  | 0.501 | *TBP* |  | 4.59 | *B2M* |  | 0.722 | *TBP* |
| 0.420 | *TBP* |  | 0.575 | *B2M* |  | 4.82 | *ACTB* |  | 0.891 | *B2M* |
| 0.791 | *B2M* |  | 0.634 | *18S* |  | 5.54 | *GAPDH* |  | 0.747 | *18S* |
| 1.068 | *GAPDH* |  | 0.790 | *GAPDH* |  | 10.21 | *18S* |  | 1.139 | *GAPDH* |
| PC samples | | | | | | | | | | |
| 0.272 | ***HPRT1*** |  | 0.545 | ***HPRT1*** |  | 2.18 | ***TBP*** |  | 0.748 | ***HPRT1*** |
| 0.492 | *TBP* |  | 0.545 | ***TBP*** |  | 2.34 | *HPRT1* |  | 0.854 | *TBP* |
| 0.606 | *B2M* |  | 0.655 | *B2M* |  | 3.07 | *GAPDH* |  | 0.899 | *B2M* |
| 0.663 | *ACTB* |  | 0.687 | *ACTB* |  | 4.20 | *ACTB* |  | 0.932 | *ACTB* |
| 0.916 | *18S* |  | 0.832 | *18S* |  | 4.39 | *B2M* |  | 1.116 | *18S* |
| 1.042 | *GAPDH* |  | 0.945 | *GAPDH* |  | 9.33 | *18S* |  | 1.189 | *GAPDH* |
| AC samples | | | | | | | | | | |
| 0.234 | ***ACTB*** |  | 0.283 | ***HPRT1*** |  | 1.89 | ***HPRT1*** |  | 0.462 | ***ACTB*** |
| 0.248 | *TBP* |  | 0.283 | ***ACTB*** |  | 1.97 | *TBP* |  | 0.478 | *TBP* |
| 0.289 | *HPRT1* |  | 0.342 | *TBP* |  | 2.30 | *GAPDH* |  | 0.480 | *HPRT1* |
| 0.421 | *B2M* |  | 0.383 | *B2M* |  | 3.08 | *ACTB* |  | 0.552 | *B2M* |
| 0.528 | *18S* |  | 0.486 | *18S* |  | 3.75 | *B2M* |  | 0.640 | *18S* |
| 0.537 | *GAPDH* |  | 0.537 | *GAPDH* |  | 6.02 | *18S* |  | 0.643 | *GAPDH* |
| CC and PC samples | | | | | | | | | | |
| 0.184 | ***HPRT1*** |  | 0.534 | ***HPRT1*** |  | 2.49 | ***TBP*** |  | 0.747 | ***HPRT1*** |
| 0.514 | *TBP* |  | 0.534 | ***TBP*** |  | 2.79 | *HPRT1* |  | 0.842 | *TBP* |
| 0.544 | *ACTB* |  | 0.644 | *B2M* |  | 4.35 | *GAPDH* |  | 0.859 | *ACTB* |
| 0.686 | *B2M* |  | 0.676 | *ACTB* |  | 4.54 | *B2M* |  | 0.914 | *B2M* |
| 0.776 | *18S* |  | 0.793 | *18S* |  | 4.68 | *ACTB* |  | 1.011 | *18S* |
| 1.043 | *GAPDH* |  | 0.916 | *GAPDH* |  | 196.43 | *18S* |  | 1.180 | *GAPDH* |
| CC and AC samples | | | | | | | | | | |
| 0.225 | ***ACTB*** |  | 0.389 | ***ACTB*** |  | 2.38 | *TBP* |  | 0.604 | ***ACTB*** |
| 0.273 | *HPRT1* |  | 0.389 | *HPRT1* |  | 2.49 | *HPRT1* |  | 0.625 | *HPRT1* |
| 0.33 | *TBP* |  | 0.430 | *TBP* |  | 3.92 | *GAPDH* |  | 0.643 | *TBP* |
| 0.563 | *18S* |  | 0.516 | *B2M* |  | 4.02 | *ACTB* |  | 0.781 | *18S* |
| 0.657 | *B2M* |  | 0.605 | *18S* |  | 4.46 | *B2M* |  | 0.794 | *B2M* |
| 0.915 | *GAPDH* |  | 0.730 | *GAPDH* |  | 8.84 | *18S* |  | 1.004 | *GAPDH* |
| All samples of cases | | | | | | | | | | |
| 0.261 | ***HPRT1*** |  | 0.481 | ***HPRT1*** |  | 2.32 | ***TBP*** |  | 0.727 | ***HPRT1*** |
| 0.454 | *ACTB* |  | 0.481 | ***TBP*** |  | 2.44 | *HPRT1* |  | 0.790 | *TBP* |
| 0.459 | *TBP* |  | 0.596 | *ACTB* |  | 3.67 | *GAPDH* |  | 0.791 | *ACTB* |
| 0.626 | *B2M* |  | 0.631 | *B2M* |  | 4.36 | *ACTB* |  | 0.862 | *B2M* |
| 0.833 | *18S* |  | 0.764 | *18S* |  | 4.54 | *B2M* |  | 1.023 | *18S* |
| 0.977 | *GAPDH* |  | 0.874 | *GAPDH* |  | 10.32 | *18S* |  | 1.112 | *GAPDH* |
| CCC samples | | | | | | | | | | |
| 0.166 | ***TBP*** |  | 0.427 | ***HPRT1*** |  | 1.91 | ***TBP*** |  | 0.760 | ***HPRT1*** |
| 0.341 | *HPRT1* |  | 0.427 | *B2M* |  | 2.59 | *HPRT1* |  | 0.780 | *TBP* |
| 0.4874 | *B2M* |  | 0.503 | *ACTB* |  | 3.18 | *B2M* |  | 0.790 | *B2M* |
| 0.5658 | *ACTB* |  | 0.587 | *TBP* |  | 3.48 | *GAPDH* |  | 0.813 | *ACTB* |
| 0.6484 | *18S* |  | 0.646 | *18S* |  | 4.26 | *ACTB* |  | 0.903 | *18S* |
| 1.393 | *GAPDH* |  | 0.890 | *GAPDH* |  | 8.87 | *18S* |  | 1.449 | *GAPDH* |
| ACC samples | | | | | | | | | | |
| 0.116 | ***TBP*** |  | 0.277 | ***HPRT1*** |  | 3.57 | ***TBP*** |  | 0.575 | ***HPRT1*** |
| 0.139 | *HPRT1* |  | 0.277 | ***TBP*** |  | 4.33 | *HPRT1* |  | 0.589 | *TBP* |
| 0.243 | *ACTB* |  | 0.377 | *B2M* |  | 5.00 | *B2M* |  | 0.614 | *ACTB* |
| 0.394 | *B2M* |  | 0.423 | *ACTB* |  | 5.20 | *GAPDH* |  | 0.660 | *B2M* |
| 0.890 | *18S* |  | 0.575 | *18S* |  | 6.27 | *ACTB* |  | 0.967 | *18S* |
| 0.999 | *GAPDH* |  | 0.721 | *GAPDH* |  | 14.26 | *18S* |  | 1.068 | *GAPDH* |
| All samples of controls | | | | | | | | | | |
| 0.137 | ***TBP*** |  | 0.443 | *B2M* |  | 2.83 | ***TBP*** |  | 0.695 | ***TBP*** |
| 0.324 | *HPRT1* |  | 0.443 | *ACTB* |  | 3.43 | *HPRT1* |  | 0.7075 | *HPRT1* |
| 0.429 | *B2M* |  | 0.485 | *HPRT1* |  | 4.49 | *B2M* |  | 0.722 | *ACTB* |
| 0.439 | *ACTB* |  | 0.523 | *TBP* |  | 4.83 | *GAPDH* |  | 0.726 | *B2M* |
| 0.739 | *18S* |  | 0.618 | *18S* |  | 5.62 | *ACTB* |  | 0.913 | *18S* |
| 1.207 | *GAPDH* |  | 0.818 | *GAPDH* |  | 11.14 | *18S* |  | 1.271 | *GAPDH* |
| CC and CCC samples | | | | | | | | | | |
| 0.279 | ***HPRT1*** |  | 0.499 | ***HPRT1*** |  | 2.55 | *TBP* |  | 0.701 | ***HPRT1*** |
| 0.341 | *ACTB* |  | 0.499 | *ACTB* |  | 3.03 | *HPRT1* |  | 0.704 | *ACTB* |
| 0.379 | *TBP* |  | 0.541 | *TBP* |  | 4.41 | *B2M* |  | 0.738 | *TBP* |
| 0.508 | *18S* |  | 0.586 | *B2M* |  | 4.78 | *ACTB* |  | 0.812 | *18S* |
| 0.715 | *B2M* |  | 0.651 | *18S* |  | 5.12 | *GAPDH* |  | 0.874 | *B2M* |
| 1.13 | *GAPDH* |  | 0.824 | *GAPDH* |  | 9.91 | *18S* |  | 1.212 | *GAPDH* |
| AC and ACC samples | | | | | | | | | | |
| 0.187 | *TBP* |  | 0.397 | *B2M* |  | 2.35 | *HPRT1* |  | 0.548 | ***ACTB*** |
| 0.190 | *ACTB* |  | 0.397 | ***ACTB*** |  | 2.48 | *TBP* |  | 0.551 | *TBP* |
| 0.401 | *B2M* |  | 0.417 | *TBP* |  | 2.95 | *GAPDH* |  | 0.625 | *B2M* |
| 0.428 | *HPRT1* |  | 0.449 | *HPRT1* |  | 3.86 | *ACTB* |  | 0.629 | *HPRT1* |
| 0.663 | *GAPDH* |  | 0.556 | *GAPDH* |  | 4.36 | *B2M* |  | 0.792 | *GAPDH* |
| 0.800 | *18S* |  | 0.661 | *18S* |  | 9.57 | *18S* |  | 0.892 | *18S* |
| All samples | | | | | | | | | | |
| 0.304 | ***HPRT1*** |  | 0.514 | ***HPRT1*** |  | 2.43 | ***TBP*** |  | 0.744 | ***HPRT1*** |
| 0.434 | *TBP* |  | 0.514 | ***TBP*** |  | 2.59 | *HPRT1* |  | 0.789 | *TBP* |
| 0.448 | *ACTB* |  | 0.607 | *ACTB* |  | 3.85 | *GAPDH* |  | 0.790 | *ACTB* |
| 0.597 | *B2M* |  | 0.627 | *B2M* |  | 4.53 | *ACTB* |  | 0.849 | *B2M* |
| 0.844 | *18S* |  | 0.759 | *18S* |  | 4.53 | *B2M* |  | 1.032 | *18S* |
| 1.010 | *GAPDH* |  | 0.879 | *GAPDH* |  | 10.53 | *18S* |  | 1.141 | *GAPDH* |

*A lower value indicates higher stability in gene expression. CC: central cuff (injured supraspinatus tendon) samples of cases; PC: posterior superior cuff samples of cases; AC: anterior cuff samples of cases; CCC: central cuff samples of controls; ACC: anterior cuff samples of controls; Bold letters: top ranked genes by more than one software.
